# Cumulative residual cholesterol predicts the risk of cardiovascular disease in the general population aged 45 years and older

**DOI:** 10.1186/s12944-023-02000-0

**Published:** 2024-01-19

**Authors:** Mengjie Zhao, Mengli Xiao, Qin Tan, Jinjin Ji, Fang Lu

**Affiliations:** 1 Xiyuan Hospital, China Academy of Chinese Medicine Sciences, Beijing, 100091 China; 2 NMPA Key Laboratory for Clinical Research and Evaluation of Traditional Chinese Medicine, Beijing, 100091 China; 3 National Clinical Research Center for Chinese Medicine Cardiology, Beijing, 100091 China

**Keywords:** Residual cholesterol, Cardiovascular disease, K-means clustering, Weighted quantile sum, CHARLS

## Abstract

**Background:**

Numerous studies have affirmed a robust correlation between residual cholesterol (RC) and the occurrence of cardiovascular disease (CVD). However, the current body of literature fails to adequately address the link between alterations in RC and the occurrence of CVD. Existing studies have focused mainly on individual RC values. Hence, the primary objective of this study is to elucidate the association between the cumulative RC (Cum-RC) and the morbidity of CVD.

**Methods:**

The changes in RC were categorized into a high-level fast-growth group (Class 1) and a low-level slow-growth group (Class 2) by K-means cluster analysis. To investigate the relationship between combined exposure to multiple lipids and CVD risk, a weighted quantile sum (WQS) regression analysis was employed. This analysis involved the calculation of weights for total cholesterol (TC), low-density lipoprotein (LDL), and high-density lipoprotein (HDL), which were used to effectively elucidate the RC.

**Results:**

Among the cohort of 5,372 research participants, a considerable proportion of 45.94% consisted of males, with a median age of 58. In the three years of follow-up, 669 participants (12.45%) had CVD. Logistic regression analysis revealed that Class 2 individuals had a significantly reduced risk of developing CVD compared to Class 1. The probability of having CVD increased by 13% for every 1-unit increase in the Cum-RC according to the analysis of continuous variables. The restricted cubic spline (RCS) analysis showed that Cum-RC and CVD risk were linearly related (*P* for nonlinearity = 0.679). The WQS regression results showed a nonsignificant trend toward an association between the WQS index and CVD incidence but an overall positive trend, with the greatest contribution from TC (weight = 0.652), followed by LDL (weight = 0.348).

**Conclusion:**

Cum-RC was positively and strongly related to CVD risk, suggesting that in addition to focusing on traditional lipid markers, early intervention in patients with increased RC may further reduce the incidence of CVD.

**Supplementary Information:**

The online version contains supplementary material available at 10.1186/s12944-023-02000-0.

## Introduction

Cardiovascular disease (CVD) stands as the foremost cause of both mortality and disability in China [1, 2]. In the past three decades, the incidence of CVD in China has risen dramatically [3]. Between 2005 and 2020, the overall burden of premature deaths from CVD in China was greater than the global average, far exceeding that in some middle- and high-income countries [4, 5]. CVD has emerged as a major public health issue that threatens people’s health and well-being. From the perspective of population epidemiology, identifying simple, economical, and reproducible indicators to establish a CVD risk prediction model has become a popular research topic in recent years and can help better identify susceptible groups at high-risk of CVD.

Dyslipidemia serves as a notable and independent risk factor in the onset and progression of cardiovascular events. For a long time, lipid-lowering targets for dyslipidemia prevention and treatment have focused mainly on low-density lipoprotein (LDL) levels. However, even when LDL reaches lipid-lowering target values, the risk of major adverse cardiovascular events remains, referred to as residual risk [6, 7]. There is growing evidence that triglyceride (TG) and/or triglyceride-rich lipoprotein (TRL) cholesterol levels may contribute to this residual risk [8]. Therefore, recent studies have gradually focused on residual cholesterol (RC). Several studies have proven the importance of RC in predicting CVD incidence and its prognosis independent of LDL [9, 10]. Monitoring RC levels may help determine potential CVD risk not reflected by LDL. Recent research [11] revealed that RC was strongly linked to the incidence of metabolic syndrome and the occurrence of CVD. This study is limited by the use of a single database and only used data from a single measurement at baseline, which may not allow us to observe trends in disease risk. Currently, most studies have focused on baseline RC levels. There are a limited number of studies examining the relationship between alterations in RC levels and CVD incidence. Baseline RC levels provide information only about static factors, while dynamically changing RC levels may better reflect an individual’s cholesterol metabolism and trends. Studying changes in RC levels can provide a better understanding of how cholesterol levels fluctuate in individuals during treatment and thus allow a more accurate assessment of the risk of CVD.

The data source is the China Health and Retirement Longitudinal Study (CHARLS) database, which contains numerous high-quality microdata encompassing the household and individual profiles of middle-aged and older adults aged 45 and older in China. Compared with previous studies that used only single measurements of RC, cumulative RC (Cum-RC) was used in this study to explore the characteristics of populations with different RC trends and to provide a holistic and comprehensive view of the impact of dynamic alterations in RC on the incidence of CVD and older to make the results more in line with real-world conditions.

### Study population

The CHARLS database provided the data for this analysis. The CHARLS national baseline survey was undertaken in 2011 (Wave 1), and further waves of the study were performed in 2013 (Wave 2), 2015 (Wave 3), and 2018 (Wave 4) in 150 counties and 450 urban and rural community neighborhood committees throughout 28 provinces. The county/district and village sampling levels used a probability proportional to size (PPS) approach. Prior to participation, all subjects provided written informed consent [12].

**Fig. 1:**
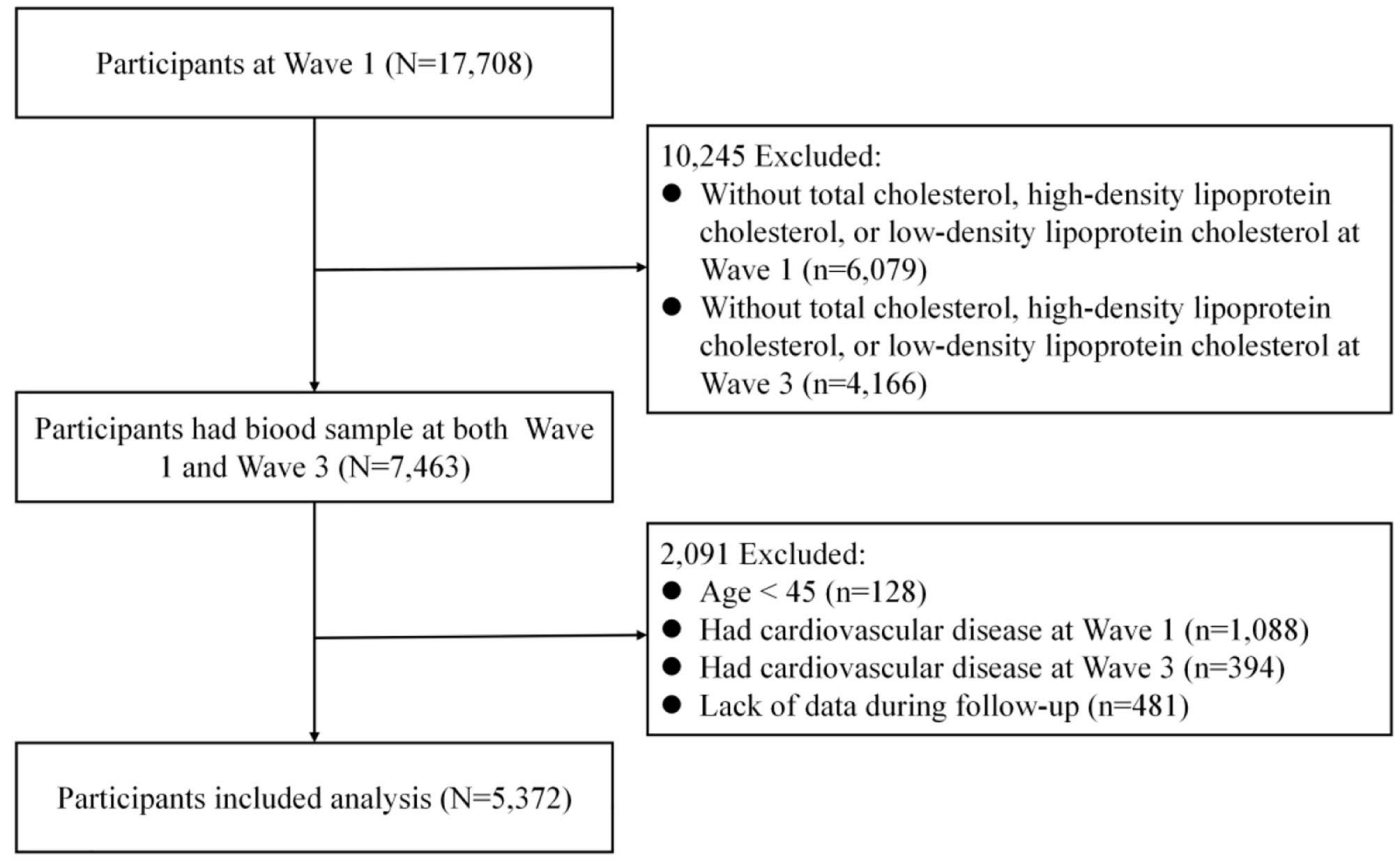
Flowchart for screening the research subjects

Data for 17,708 study subjects were collected from the 2011 baseline survey as the initial population, and those who met the study objectives were selected according to the following inclusion criteria: (1) aged ≥ 45 at Wave 1; (2) had total cholesterol (TC), high-density lipoprotein (HDL), and LDL levels at Wave 1 and Wave 3; (3) had not yet suffered from CVD, including heart disease and stroke, at Wave 1 and Wave 3; and (4) had CVD status information recorded at Wave 4. Ultimately, 5,372 patients were included in the study population. Figure 1 depicts the specific filtering procedure for respondents.

### Evaluation of RC and Cum-RC

The Cum-RC values from 2012 to 2015 were used for this study. The formula RC = TC – HDL – LDL [13] was used to calculate the RC values. In addition, the cumulative level of RC between 2012 and 2015 was calculated based on the formula Cum-RC = (RC_2012_ + RC_2015_)/2 × time (2015 − 2012) [14].

### Definition of CVD

New-onset CVD was the primary outcome of this research. According to a previous CHARLS-related study, CVD incidence was ascertained by the patient’s answers to the questionnaire question in Wave 4: “Has your doctor ever told you that you have a heart-related illness (including angina, myocardial infarction, coronary artery disease, congestive heart failure, or other heart disease) or had a stroke?” If the participant answered “yes”, he or she was defined as having experienced a cardiovascular event [15, 16].

### Covariates

Baseline data were collected from in-person interviews of study participants by staff trained in questionnaire administration. The questionnaire covered demographic information (age, sex, residence, marriage), body mass index (BMI), health status (hypertension, dyslipidemia, and diabetes), lifestyle information (smoking, alcohol consumption), medication use (antihypertensive, lipid-lowering, hypoglycemic), and socioeconomic status (education). The residences were categorized as urban or rural [12]. Disease history included hypertension, dyslipidemia, and diabetes. In terms of education level, the study participants were categorized into four groups: no education, primary education, secondary education, and college education and above. Fasting venous blood collection was performed by professionally trained personnel, and TC, LDL, HDL, FPG, glycosylated hemoglobin A1c (HbA1c), and uric acid (UA) were measured.

### Statistical analysis

Given the non-normal distribution of the study measurements, they were reported using the median and interquartile range (IQR), and counts were statistically described using frequency and percentage [n (%)]. Multiple imputations were used to fill in missing data to maximize statistical power and mitigate any bias that may result from missing data [17]. Information on missing variables can be found in Table S1. When grouping Cum-RCs, two methods, namely, K-means clustering and tertile grouping, were tested. To investigate the relationship between Cum-RC and the development of new-onset CVD, a logistic regression model was employed, adjusting for potential confounding factors. The K-means clustering algorithm is an iterative technique employed to cluster data by utilizing distance as a measure of similarity in order to divide a given dataset into K distinct classes. In the clustering process, each class is characterized by a clustering center, which is determined by calculating the mean value of all the data points within that particular class [18, 19].

The process of K-means clustering can be briefly summarized in the following steps. First, a collection of k data points is randomly selected from the dataset to serve as the initial clustering centers. Then, all the data points in the dataset are traversed and each data point is assigned to the category corresponding to the cluster center nearest to it. Next, the clustering centers for each category are recalculated, and a new category is derived by calculating the mean value of all the data points within the class. The above steps are repeated until all the data points reach the minimum sum of the distances to the clustering centers of the classes to which they belong [20].

In this paper, the clustering effect was evaluated through the silhouette coefficient, which dynamically determines the range of values of K. The silhouette coefficient was first proposed by Peter J. Rousseeuw in 1986 based on the comparison of closeness and separation and can be used to choose an optimal number of clusters and provide an assessment of clustering effectiveness [21]. The silhouette coefficients, ranging from − 1 to + 1, provide a measure of the similarity between sample points and their respective clusters, with values closer to 1 suggesting a strong fit within their assigned clusters and a weaker fit with neighboring clusters [22]. In the evaluation process, each profile coefficient corresponds to a specific value of K. Therefore, a reasonable range of values of K can be determined based on the higher profile coefficients. The silhouette coefficient relationship graph (Fig. S1) shows that the clustering effect is optimal when K is 2.

**Fig. 2:**
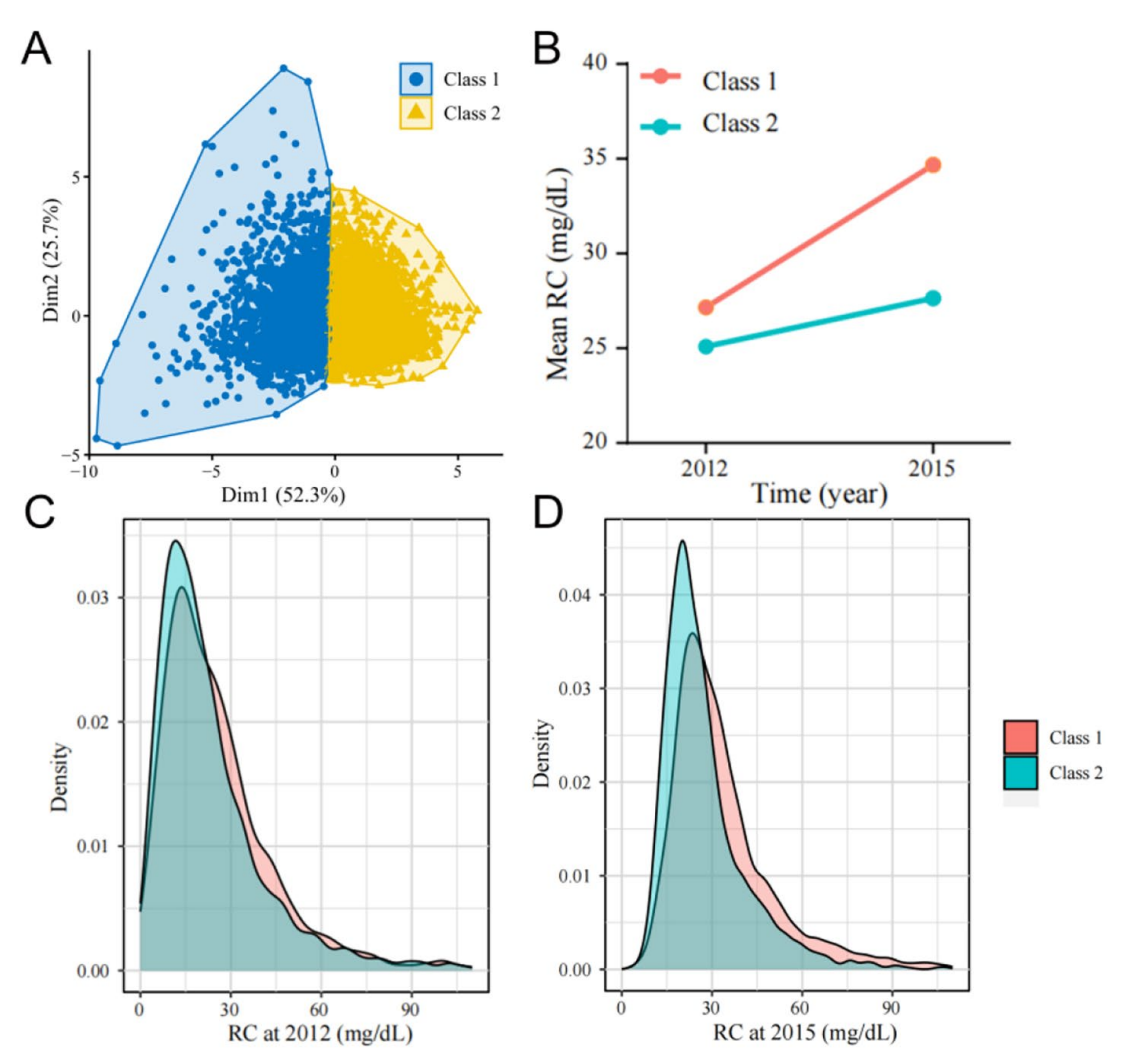
Analysis of changes in RC via the K-means clustering model. **(A)** Scatterplot visualizing the distribution of two categories of data based on K-means clustering; **(B)** trend of RC change in two categories of population after clustering; **(C)** density plot of RC distribution in 2012 for two categories of population; **(D)** density plot of RC distribution in 2015 for two categories of population

Figure 2A shows how the clustered population was divided. In Fig. 2B, for the Class 1 (*n* = 2,219) population, the RC range increased from 22.04 (12.76, 34.02) mg/dL × years in 2012 to 29.73 (22.39, 39.96) mg/dL × years in 2015 (*P* < 0.001), with a Cum-RC of 78.82 (57.94, 108.90) mg/dL × years; additionally, the RC showed a rapid increasing trend. For the Class 2 (*n* = 3,153) group, the RC range increased from 18.56 (10.82, 30.54) mg/dL × years in 2012 to 23.94 (18.15, 32.05) mg/dL × years in 2015 (*P* < 0.001), with a Cum-RC of 65.06 (47.51, 92.71) mg/dL × years, and the RC showed a slow increasing trend. Moreover, Fig. 2C and D show the distributions of RCs in the Class 1 and Class 2 groups, respectively, and reveal the differences between the two groups. Notably, these data all exhibited a nonnormal distribution.

RCS modeling was used to explore the dose-response relationship between the Cum-RC and CVD risk. Subgroup analyses were performed. In addition, an analysis was performed to determine whether there was an interaction effect between these risk factors and the Cum-RC on the development of CVD and test for trends in exposure levels in different subgroups.

In addition, a weighted quantile sum (WQS) regression model was utilized to explore the overall association between exposure to the three lipids (TC, LDL, and HDL) among the RC components and CVD risk and to establish the relative contribution of each lipid to CVD risk [23]. In the WQS regression, the weight values for exposure range from 0 to 1, and the sum of the weights is 1 [24]. A higher weight value indicates a greater degree of contribution of the component exposure to the overall load. WQS regression was used to assess the association between combined exposure to the three lipids as a whole and CVD risk. The exposure level of each lipid was converted into an ordinal variable that was weighted and summed in interquartile form to obtain the sum of the weighted quartiles of all exposure elements (WQS index). The WQS index represents the overall exposure load of the three lipids and was combined with the covariates above in a regression model reflecting the effect of combined exposure on outcome [25].

The statistical analyses were completed using Stata 16.0 and R 4.1.1 software.

## Results

### Baseline characteristics

There were 5,372 people in this study, with a median age of 58 years. A total of 45.94% of the participants were male, 53.02% had completed elementary school and above, and 47.82% were rural dwellers. Based on data from 2012 to 2015, the medians (IQRs) for RC were 19.72 (11.60, 32.09) mg/dL × years and 25.87 (19.69, 35.91) mg/dL × years, respectively. The median (IQR) for Cum-RC was 70.13 (51.55, 100.30) mg/dL × years. Compared to those in the Class 1 group, the Class 2 participants were younger; had a greater proportion of males, smokers, and alcoholics; were more educated; had a lower BMI, systolic blood pressure (SBP), and diastolic blood pressure (DBP); had a lower incidence of hypertension and dyslipidemia; and had lower levels of FPG, Hba1c, UA, TC, HDL, LDL, and Cum-RC (*P* < 0.05). In addition, tertiles of Cum-RC levels were used to divide participants into 3 groups (Table 1). Compared with those in the first tertile (T1), participants in the third tertile (T3) were younger; had a greater proportion of females and nonsmokers; had a greater BMI; and had a greater incidence of hypertension, dyslipidemia, and diabetes (*P* < 0.001). In addition, increased Cum-RC was positively correlated with SBP, DBP, FPG, HbA1c, UA, TC, LDL, RC_2012_, and RC_2015_ and negatively correlated with HDL (*P* < 0.001).

**Table 1:** Baseline characteristics

Variables		Cum-RC exposure		***P***-value	Cum-RC exposure			***P***-value
	Total	Class 1	Class 2		T1 (≤ 57.36)	T2 (57.36–87.52)	T3 (> 87.52)	
N	5372	2219	3153		1792	1789	1791	
Age, years	58.00 (51.00, 64.00)	58.00 (53.00, 64.00)	57.00 (51.00, 63.00)	< 0.001	58.00 (52.00, 64.00)	58.00 (52.00, 65.00)	57.00 (51.00, 62.00)	< 0.001
Sex, n (%)				< 0.001				< 0.001
Male	2468 (45.94)	828 (37.31)	1640 (52.01)		958 (53.46)	808 (45.16)	702 (39.20)	
Residence, n (%)				0.380				0.113
Rural	2569 (47.82)	1077 (48.54)	1492 (47.32)		821 (45.81)	872 (48.74)	876 (48.91)	
Urban	2803 (52.18)	1142 (51.46)	1661 (52.68)		971 (54.19)	917 (51.26)	915 (51.09)	
Education level, n (%)				0.002				0.579
No formal education	2524 (46.98)	1087 (48.99)	1437 (45.58)		847 (47.27)	846 (47.29)	831 (46.40)	
Primary school	1227 (22.84)	519 (23.39)	708 (22.45)		413 (23.05)	412 (23.03)	402 (22.45)	
Middle or high school	1478 (27.51)	549 (24.74)	929 (29.46)		492 (27.46)	487 (27.22)	499 (27.86)	
College or above	143 (2.66)	64 (2.88)	79 (2.51)		40 (2.23)	44 (2.46)	59 (3.29)	
Marital status, n (%)				0.892				0.095
Married	4855 (90.38)	2004 (90.31)	2851 (90.42)		1604 (89.51)	1611 (90.05)	1640 (91.57)	
Other	517 (9.62)	215 (9.69)	302 (9.58)		188 (10.49)	178 (9.95)	151 (8.43)	
Smoking status, n (%)				< 0.001				< 0.001
Yes	2050 (38.16)	700 (31.55)	1350 (42.82)		762 (42.52)	685 (38.29)	603 (33.67)	
Drinking status, n (%)				0.007				0.176
Yes	1225 (22.80)	465 (20.96)	760 (24.10)		435 (24.28)	400 (22.36)	390 (21.78)	
BMI, kg/m^2^, n (%)				< 0.001				< 0.001
18.5–24	2847 (53.00)	1118 (50.38)	1729 (54.84)		1134 (63.28)	993 (55.51)	720 (40.20)	
< 18.5	325 (6.05)	116 (5.23)	209 (6.63)		169 (9.43)	106 (5.93)	50 (2.79)	
24–28	1590 (29.60)	708 (31.91)	882 (27.97)		393 (21.93)	499 (27.89)	698 (38.97)	
> 28	610 (11.36)	277 (12.48)	333 (10.56)		96 (5.36)	191 (10.68)	323 (18.03)	
Hypertension, n (%)				0.001				< 0.001
Yes	1134 (21.11)	516 (23.25)	618 (19.60)		277 (15.46)	371 (20.74)	486 (27.14)	
Dyslipidemia, n (%)				0.002				< 0.001
Yes	413 (7.69)	200 (9.01)	213 (6.76)		86 (4.80)	122 (6.82)	205 (11.45)	
Diabetes, n (%)				0.633				< 0.001
Yes	255 (4.75)	109 (4.91)	146 (4.63)		56 (3.12)	86 (4.81)	113 (6.31)	
Lipid-lowering drugs, n (%)				0.282				< 0.001
Yes	228 (4.24)	102 (4.60)	126 (4.00)		44 (2.46)	67 (3.75)	117 (6.53)	
Antihypertensive drugs, n (%)				< 0.001				< 0.001
Yes	828 (15.41)	389 (17.53)	439 (13.92)		182 (10.16)	280 (15.65)	366 (20.44)	
Hypoglycemic drugs, n (%)				0.660				< 0.001
Yes	170 (3.16)	73 (3.29)	97 (3.08)		34 (1.90)	60 (3.35)	76 (4.24)	
SBP, mmHg	127.30 (115.70, 141.00)	129.00 (117.00, 142.30)	126.30 (114.70, 140.10)	< 0.001	124.70 (113.30, 138.60)	128.00 (116.30, 141.00)	129.90 (117.70, 143.30)	< 0.001
DBP, mmHg	75.00 (68.06, 82.67)	75.33 (68.67, 83.05)	74.37 (67.93, 82.27)	0.003	73.67 (66.67, 80.82)	74.88 (68.25, 82.40)	76.56 (69.67, 84.67)	< 0.001
FPG	102.40 (94.50, 112.50)	103.70 (95.76, 113.40)	101.30 (93.96, 111.60)	< 0.001	99.72 (92.88, 107.60)	101.30 (93.96, 110.50)	106.70 (98.01, 120.40)	< 0.001
HbA1c, %	5.10 (4.90, 5.40)	5.20 (4.90, 5.50)	5.10 (4.80, 5.40)	< 0.001	5.10 (4.80, 5.30)	5.10 (4.90, 5.40)	5.20 (4.90, 5.50)	< 0.001
UA, mg/dL	4.23 (3.52, 5.07)	4.30 (3.57, 5.15)	4.18 (3.49, 5.01)	< 0.001	4.05 (3.42, 4.80)	4.21 (3.49, 5.04)	4.43 (3.69, 5.34)	< 0.001
TC_2012_, mg/dL	190.60 (167.40, 214.90)	219.60 (204.10, 240.50)	171.70 (155.80, 187.10)	< 0.001	178.60 (158.50, 202.20)	189.80 (168.20, 212.20)	202.60 (178.00, 230.60)	< 0.001
HDL_2012_, mg/dL	49.48 (40.21, 59.92)	51.80 (42.91, 62.24)	47.94 (38.66, 57.99)	< 0.001	56.44 (48.61, 67.27)	50.64 (42.91, 59.92)	40.98 (34.02, 49.10)	< 0.001
LDL_2012_, mg/dL	114.00 (93.56, 136.90)	141.50 (126.40, 158.50)	98.58 (83.12, 112.90)	< 0.001	110.60 (92.78, 130.30)	116.00 (96.26, 137.60)	116.00 (91.24, 143.00)	< 0.001
RC_2012_, mg/dL	19.72 (11.60, 32.09)	22.04 (12.76, 34.02)	18.56 (10.82, 30.54)	< 0.001	10.05 (6.57, 13.53)	20.49 (15.46, 25.90)	39.43 (29.00, 53.74)	< 0.001
RC_2015_, mg/dL	25.87 (19.69, 35.91)	29.73 (22.39, 39.96)	23.94 (18.15, 32.05)	< 0.001	18.92 (15.44, 22.39)	26.25 (21.62, 31.27)	41.31 (32.05, 53.28)	< 0.001
Cumulative RC, mg/dL × years	70.13 (51.55, 100.30)	78.82 (57.94, 108.90)	65.06 (47.51, 92.71)	< 0.001	45.20 (37.67, 51.55)	70.13 (63.74, 77.67)	119.40 (100.30, 152.40)	< 0.001

### Associations between Cum-RC scores and new-onset CVD incidence

**Fig. 3:**
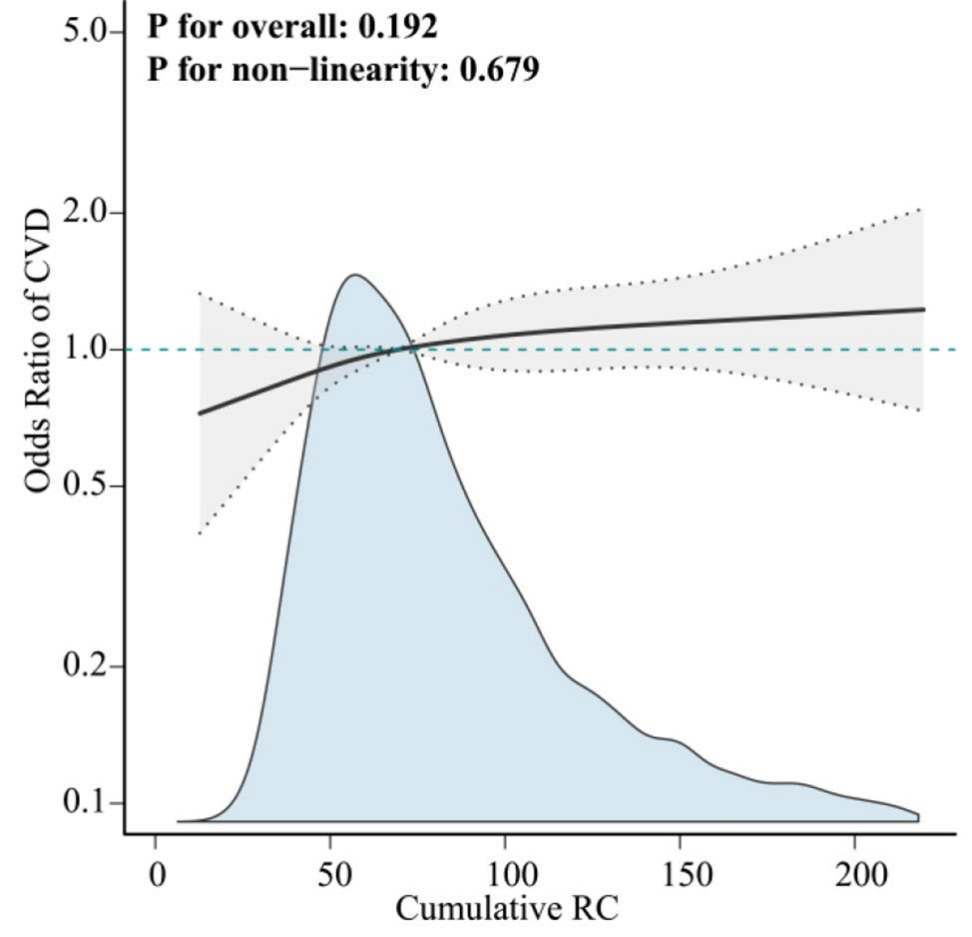
Linear associations between Cum-RC and CVD incidence. We adjusted for potential confounders, including age, sex, education level, marital status, residence, BMI, smoking status, drinking status, SBP, DBP, hypertension, dyslipidemia, diabetes, lipid-lowering drugs, antihypertensive drugs, hypoglycemic drugs, FPG, HbA1c, and UA

Table 2 shows that after 3 years of follow-up, 669 participants (12.45%) developed CVD, 407 (7.58%) had heart disease, and 300 (5.58%) had stroke. There was a lower risk of CVD in the Class 2 subgroup than in the Class 1 subgroup, with a lower risk of heart disease; moreover, the risk of stroke occurrence did not significantly differ. In addition, a comparison of T3 with T1 in the Cum-RC cohort revealed a risk of CVD (OR = 1.27, 95% CI = 1.02–1.58), heart disease (OR = 1.17, 95% CI = 0.90–1.54), and stroke (OR = 1.51, 95% CI = 1.09–2.07). Notably, Cum-RC was significantly associated with an elevated risk of CVD and stroke (*P* for trend = 0.033 and 0.012, respectively). However, the increase in the risk of developing heart disease was nonsignificant (*P* for trend = 0.249). Notably, in the RCS regression model, a positive linear correlation between Cum-RC and CVD risk was observed (*P* for nonlinearity = 0.679) (Fig. 3).

**Table 2:** The association between Cum-RC and CVD incidence status

	No. Events (%)	Crude	***P***	Model 1	***P***	Model 2	***P***	Model 3	***P***
**Cardiovascular disease**									
**Cum-RC**									
Class 1	315 (14.20)	1		1		1		1	
Class 2	354 (11.29)	0.76 (0.65,0.90)	0.001	0.79 (0.67,0.93)	0.004	0.81 (0.68,0.95)	0.011	0.80 (0.68,0.95)	0.011
**Cum-RC**									
≤ 57.36	185 (10.32)	1		1		1		1	
57.36–87.52	229 (12.80)	1.28 (1.04,1.57)	0.021	1.26 (1.02,1.55)	0.030	1.20 (0.97,1.47)	0.094	1.18 (0.96,1.45)	0.125
> 87.52	255 (14.24)	1.44 (1.18,1.76)	< 0.001	1.45 (1.18,1.78)	< 0.001	1.30 (1.05,1.61)	0.015	1.27 (1.02,1.58)	0.032
*P* for trend		< 0.001		< 0.001		0.016		0.033	
**Heart disease**									
**Cum-RC**									
Class 1	202 (9.10)	1		1		1		1	
Class 2	205 (6.54)	0.76 (0.65,0.90)	0.001	0.80 (0.68,0.95)	0.011	0.73 (0.60,0.90)	0.003	0.75 (0.61,0.92)	0.005
**Cum-RC**									
≤ 57.36	117 (6.53)	1		1		1		1	
57.36–87.52	140 (7.83)	1.22 (0.94,1.57)	0.133	1.17 (0.91,1.51)	0.224	1.13 (0.87,1.46)	0.358	1.13 (0.87,1.46)	0.370
> 87.52	150 (8.38)	1.31 (1.02,1.68)	0.036	1.25 (0.97,1.61)	0.085	1.15 (0.88,1.49)	0.307	1.17 (0.90,1.54)	0.245
*P* for trend		0.037		0.088		0.315		0.249	
**Stroke**									
**Cum-RC**									
Class 1	134 (6.04)	1		1		1		1	
Class 2	166 (5.30)	0.86 (0.68,1.09)	0.224	0.85 (0.67,1.09)	0.198	0.89 (0.70,1.13)	0.334	0.89 (0.70,1.14)	0.359
**Cum-RC**									
≤ 57.36	75 (4.19)	1		1		1		1	
57.36–87.52	101 (5.65)	1.37 (1.01,1.86)	0.044	1.39 (1.02,1.89)	0.036	1.30 (0.95,1.77)	0.098	1.27 (0.93,1.74)	0.132
> 87.52	124 (6.92)	1.70 (1.27,2.29)	< 0.001	1.84 (1.37,2.48)	< 0.001	1.61 (1.18,2.19)	0.003	1.51 (1.09,2.07)	0.012
*P* for trend		< 0.001		< 0.001		0.003		0.012	

Crude: unadjusted; Model 1: corrected for age, sex, education level, marital status and residence; Model 2: Model 1 + BMI, smoking status, drinking status, SBP and DBP; Model 3: Model 2 + hypertension, dyslipidemia, diabetes, lipid-lowering drugs, antihypertensive drugs, hypoglycemic drugs, FPG, HbA1c, and UA

### Subgroup analysis

In subgroup analyses, an interaction between Cum-RC and age as well as hypertension was found (Table 3). Among participants who were < 60 years of age, female, lived in rural areas, married, had a BMI < 24 kg/m^2^, did not smoke, did not drink alcohol, were not hypertensive, had normal lipids, or did not have diabetes, Class 2 was associated with a lower risk of CVD (*P* < 0.05) (Table 3). In addition, a subgroup analysis of Cum-RC tertiles was performed and did not reveal an interaction between Cum-RC and subgroup variables. However, among participants who were male, lived in rural areas, smoked, drank alcohol, had a BMI < 24 kg/m^2^ and were free of dyslipidemia, the risk of CVD increased with increasing Cum-RC (*P* < 0.05) (Table S2).

**Table 3:** Subgroup analysis based on clustering results

Subgroup	N	Cum-RC, OR (95%CI)		***P*** value	***P***_interaction_
		Class 1	Class 2		
Age					0.047
< 60	3145	1	0.69 (0.54, 0.87)	0.002	
≥ 60	2227	1	1.00 (0.78, 1.28)	0.993	
Sex					0.676
Male	2468	1	0.85 (0.66, 1.11)	0.227	
Female	2904	1	0.78 (0.63, 0.98)	0.034	
Residence					0.108
Rural	2569	1	0.74 (0.58, 0.94)	0.013	
Urban	2803	1	0.88 (0.70, 1.12)	0.311	
Marital status					0.548
Married	4855	1	0.81 (0.67, 0.96)	0.016	
Other	517	1	0.85 (0.48, 1.50)	0.570	
Smoke					0.243
Yes	2050	1	0.92 (0.70, 1.22)	0.563	
Never	3322	1	0.74 (0.60, 0.91)	0.006	
Drink					0.624
Yes	1225	1	0.85 (0.60, 1.21)	0.363	
Never	4147	1	0.79 (0.65, 0.96)	0.019	
BMI					0.566
< 24	3172	1	0.78 (0.62, 0.98)	0.035	
≥ 24	2200	1	0.84 (0.66, 1.08)	0.170	
Hypertension					0.002
Yes	1134	1	1.24 (0.91, 1.69)	0.182	
Never	4238	1	0.67 (0.55, 0.82)	< 0.001	
Dyslipidemia					0.255
Yes	413	1	1.13 (0.66, 1.91)	0.663	
Never	4959	1	0.79 (0.66, 0.94)	0.009	
Diabetes					0.650
Yes	255	1	1.22 (0.59, 2.51)	0.592	
Never	5117	1	0.79 (0.67, 0.94)	0.008	

In multivariate models, potential confounders other than grouping variables were adjusted for, including age, sex, education level, marital status, residence, BMI, smoking status, drinking status, SBP, DBP, hypertension, dyslipidemia, diabetes, lipid-lowering drugs, antihypertensive drugs, hypoglycemic drugs, FPG, HbA1c, and UA

### Joint lipid exposure analysis based on WQS analysis

**Fig. 4:**
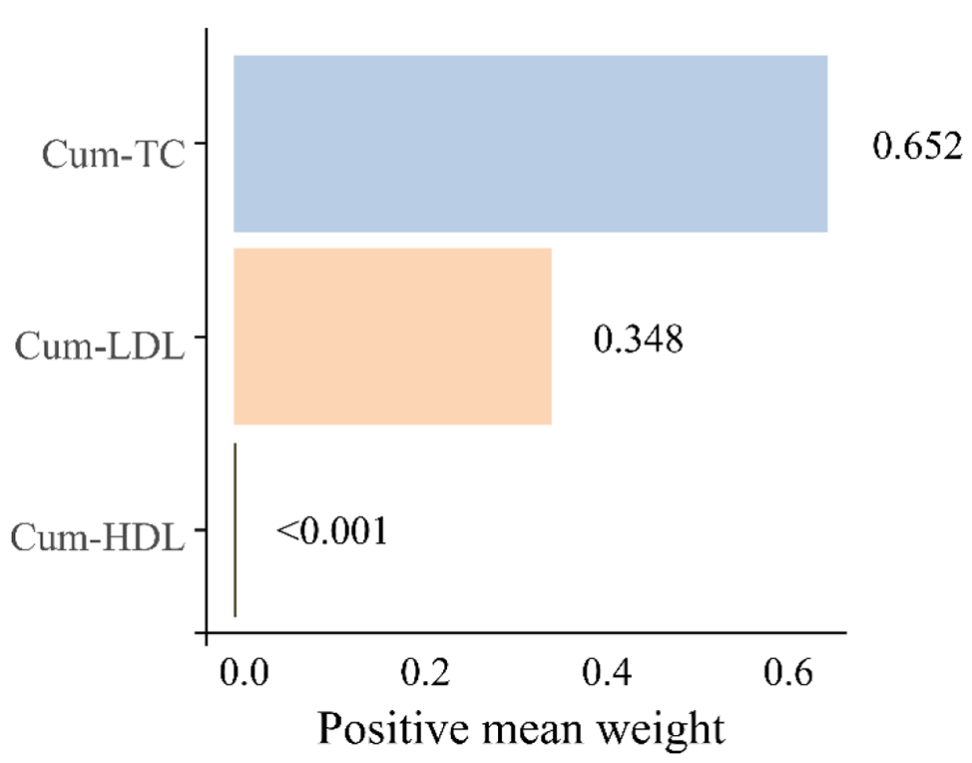
Estimated weights of the three lipids for CVD. We adjusted for age, sex, education level, marital status, residence, BMI, smoking status, drinking status, SBP, DBP, hypertension, dyslipidemia, diabetes, lipid-lowering drugs, antihypertensive drugs, hypoglycemic drugs, FPG, HbA1c, and UA

**Fig. 5:**
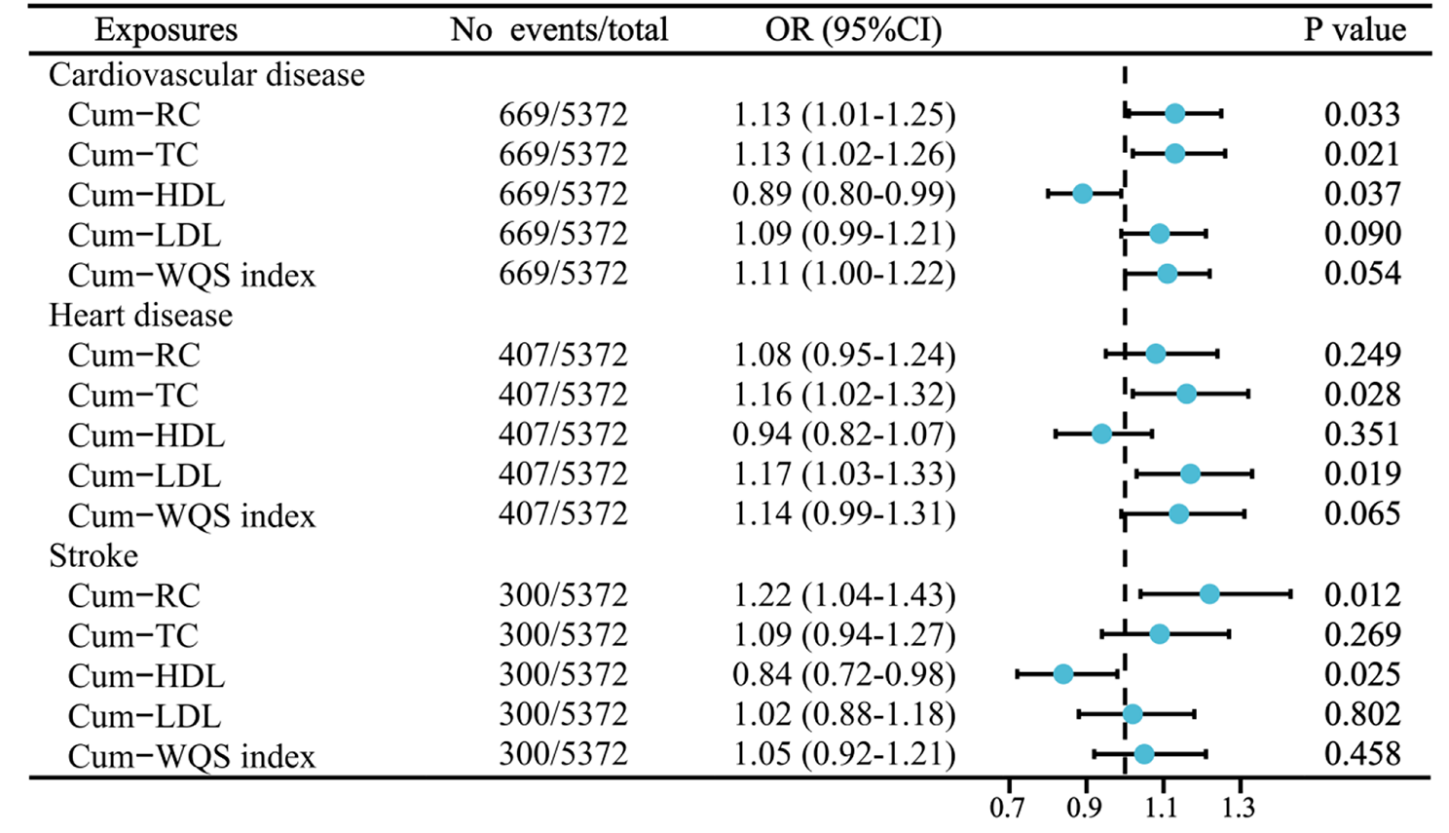
WQS modeling to analyze the association between combined exposure to three lipids and CVD risk. We adjusted for age, sex, education level, marital status, residence, BMI, smoking status, drinking status, SBP, DBP, hypertension, dyslipidemia, diabetes, lipid-lowering drugs, antihypertensive drugs, hypoglycemic drugs, FPG, HbA1c, and UA

An in-depth analysis of TC, LDL, and HDL levels in the Cum-RC was performed using the WQS regression model. The model assessed the association of cumulative TC (Cum-TC), cumulative LDL (Cum-LDL), and cumulative HDL (Cum-HDL) exposures with CVD risk. The WQS regression results showed that Cum-TC had the highest relative contribution weight (0.652) among the three variables, followed by Cum-LDL (Fig. 4). Although the effect of the WQS index of mixed lipids of TC, LDL, and HDL on CVD incidence was nonsignificant (OR = 1.11, 95% CI = 1.00–1.22, *P* > 0.05), the confidence interval did not cross 1, with an overall positive trend (Fig. 5).

However, the WQS index of mixed lipids was associated with heart disease and stroke risk (OR = 1.14, 95% CI = 0.99–1.31; OR = 1.05, 95% CI = 0.92–1.21), with confidence intervals spanning 1; moreover, the association was not significant. In addition, there was a strong correlation between Cum-TC and the risk of CVD and heart disease (*P* < 0.05) (Fig. 5). Cum-LDL was associated with heart disease alone (*P* < 0.05). In addition, Cum-HDL was inversely connected with the risk of CVD and stroke (*P* < 0.05).

### Sensitivity analyses

A regression analysis was performed after excluding participants with extreme BMI (< 18.5 or > 30 kg/m^2^) and dyslipidemia. The results showed that all outcomes remained virtually unchanged after excluding these participants. A statistically significant (*P* < 0.05) correlation was shown between high Cum-RC and increased CVD risk in both the clustered and tertile groups. Similarly, Class 2 patients had a significantly lower risk of heart disease than Class 1 patients (*P* < 0.05). The T3 group exhibited a significantly higher risk of stroke (*P* < 0.05) compared to the T1 group (Tables S3). However, no significant alteration in the risk of heart disease was observed (*P* > 0.05) (Table S4).

## Discussion

The present investigation examined the relationship between Cum-RC and CVD risk by utilizing two distinct statistical analysis models. Cum-RC exhibited an independent association with CVD risk among individuals aged 45 years and older in the CHARLS database. The WQS model demonstrated a mixed effect of combined TC, LDL, and HDL exposures on outcomes, and the WQS index tended to correlate positively with the risk of CVD, with TC contributing the most.

Ideal lipid levels are essential for reducing cardiovascular-related risks. Currently, LDL is the primary target for assessing and treating atherosclerotic cardiovascular disease (ASCVD) risk, whereas non-HDL-C or apolipoprotein B (Apo B) are considered secondary targets [26, 27]. Although LDL is a common biomarker used to assess the reduction in ASCVD risk, RC has attracted increasing amounts of attention in recent years due to its potential to trigger endothelial damage and atherosclerosis with less modification than LDL [28].

Many recent studies have shown that RC has a substantial impact on CVD risk and prognostic outcomes [13, 29–31]. The results of this analysis suggested that lower Cum-RC was linked to a lower risk of CVD. When considering LDL, however, there was no such correlation. These results are consistent with those of prior research [32, 33]. A cohort study of Spanish older adults revealed that cardiovascular outcomes were associated with TG and RC levels but not with LDL [32]. These results were further confirmed by a substantial prospective cohort study performed in Canada [33]. Furthermore, a Korean cohort study showed that RC had a marginally greater impact on CVD incidence than LDL and that high RC in combination with LDL posed a greater risk of CVD than either indication alone [34]. A recent Chinese longitudinal cohort study also revealed that RC was more independently linked with atherosclerosis progression than was LDL [35]. All these studies provide evidence that RC has a bearing on CVD incidence and suggest that combining RC/RC with LDL may be superior to LDL alone as an early assessment tool for CVD incidence. Therefore, RC is hypothesized to potentially become one of the primary targets for lipid-lowering treatments. Recent ASCVD prevention guidelines also recommend using non-HDL rather than LDL alone [26, 36]. Multiple meta-analyses [37, 38] have underscored the significance of incorporating RC as a potential biomarker in the evaluation and prediction of CVD risk and adverse cardiovascular events. Prior studies have indicated a positive association between elevated RC and an elevated risk of ASCVD in diabetic patients [39].

However, this study revealed no evidence of a link between Cum-RC and CVD risk in diabetes patients following stratified analysis. The inconsistency between the results of these two studies may be related to factors such as differences in definitions of exposures and outcomes and the national and ethnic heterogeneity of the study populations. In addition, adjustment for glucose-lowering medications may have been an influential factor. Importantly, in the subgroup analyses of Cum-RC data based on tertile groupings, there was no evidence of a connection between Cum-RC and CVD risk in either the hypertension cohort or the nonhypertensive population. A study from the CHARLS cohort showed a significant effect of increased RC on CVD incidence in the hypertensive population. However, no such association was observed among the group without hypertension [40]. That study used only a single measurement of RC, which may be the main reason for its inconsistency with the results of the present study. Finally, in the subgroup analysis based on clustered grouped data, in the nonhypertensive population, CVD risk was significantly lower in the low-level slow-growth RC group (Class 2) than in the high-level fast-growth RC group (Class 1) (*P* < 0.001). The difference in results was strongly associated with the grouping method. A likely explanation is that clustering models can combine multiple variables and group datasets at multiple time points and dimensions to better understand each subgroup’s characteristics and differences. In contrast, considering only one variable, RC, and dividing the intervals according to quartiles did not characterize each subgroup.

RC represents the cholesterol composition within TRLs [41]. There are several explanations for the mechanism by which RC contributes to ASCVD. First, RC can reach the arterial intima at a slower rate than LDL [42]. After some TG are broken down, cholesterol builds up in the intima, leading to plaque formation and the development of ASCVD [43]. Second, RC is the major oxidized lipoprotein in plasma and does not require oxidation in vitro but can be as pro-inflammatory and pro-ASCVD as LDL [44]. In addition, RC can cause low-grade inflammation [45]. The underlying mechanism may be because lipid lipases on the surface of RC residues lead to the release of free fatty acids, monoacylglycerols, and other molecules, all of which may contribute to localized damage and inflammation [46]. High levels of RC may be associated with arterial wall inflammation following endothelial injury, and persistent inflammatory stimuli may lead to hyperproliferation of vascular smooth muscle cells and neointimal hyperplasia [45, 47].

### Strengths and limitations

This study has several advantages. First, compared with previous RC-CVD association studies in which only single measurements were performed, the study used cumulative exposures for the analysis, thus increasing the reliability of the findings. Second, the WQS joint exposure model was created to assess complex human exposure patterns and actual exposure levels. The WQS model can evaluate the combined impact of several lipid components on CVD incidence risk and assign a relative importance weight to each lipid. It is more sensitive than single lipid models for identifying risk factors.

Several limitations should be acknowledged in this study. First, the study used calculated RC levels rather than direct measurements due to database limitations. Although calculated RC concentrations may introduce a degree of bias, it has been shown that calculated RC concentrations correlate well with direct measurements [48]. Moreover, the European Atherosclerosis Society Consensus Statement advocates for the combined utilization of directly measured and calculated RC data in clinical practice [46]. Currently, indirect computation methods are commonly employed in most studies because of their economic convenience and time efficiency [13, 32, 49]. Second, because individuals without complete TC, LDL, or HDL data were excluded, selection bias may have been introduced, whereby missing data are associated with specific characteristics that are also associated with study outcomes. This could lead to underestimation or overestimation of true associations. In addition, there may be information bias due to incomplete or inaccurate data, which can affect the precision of the estimates and potentially distort the observed relationships between variables. Third, because only two blood tests were performed, more detailed information on the development of RC levels could not be obtained. Fourth, caution should be exercised when extrapolating the results of this study, as it exclusively involved participants aged 45 years and older from the Chinese population.

## Conclusion

The study revealed a noteworthy association between elevated RC levels and heightened CVD risk among middle-aged and elderly individuals in the Chinese population. Specifically, the Class 1 group - characterized by a high level of rapidly increasing RC - exhibited a considerably heightened susceptibility to developing CVD. This study posited Cum-RC as a potential predictor of CVD risk, based on the observed outcomes. Aggressive RC interventions and more frequent cardiovascular monitoring appear to be necessary for high-risk patients.

### Electronic supplementary material

Below is the link to the electronic supplementary material.

Supplementary Material 1

## Abbreviations

CVD: Cardiovascular disease
RC: Remnant cholesterol
CHARLS: China health and retirement longitudinal study
Cum-RC: Cumulative remnant cholesterol
RCS: Restricted cubic spline
WQS: Weighted quantile sum
TC: Total cholesterol
LDL: Low-density lipoprotein
HDL: High-density lipoprotein
OR: Odds ratio
CI: Confidence interval
TRLs: Triglyceride rich lipoproteins
PPS: Probability proportional to size
BMI: Body mass index
SBP: Systolic blood pressure
DBP: Diastolic blood pressure
TG: Triglycerides
FPG: Fasting plasma glucose
HbA1c: Glycated hemoglobin A1c
UA: Uric acid
T1-T3: Tertile 1-Tertile 3
IQR: Interquartile range
ASCVD: Atherosclerotic cardiovascular disease
Apo B: Apolipoprotein B
