# Supplementary material for: Cumulative residual cholesterol predicts the risk of cardiovascular disease in the general population aged 45 years and older
Source: Lipids Health Dis. 2024 Jan 19;23:19. doi: 10.1186/s12944-023-02000-0 (PMC10797780; doi:10.1186/s12944-023-02000-0)
Supplement: Supplementary file 1 — Supplementary Material 1 [file 12944_2023_2000_MOESM1_ESM.docx]

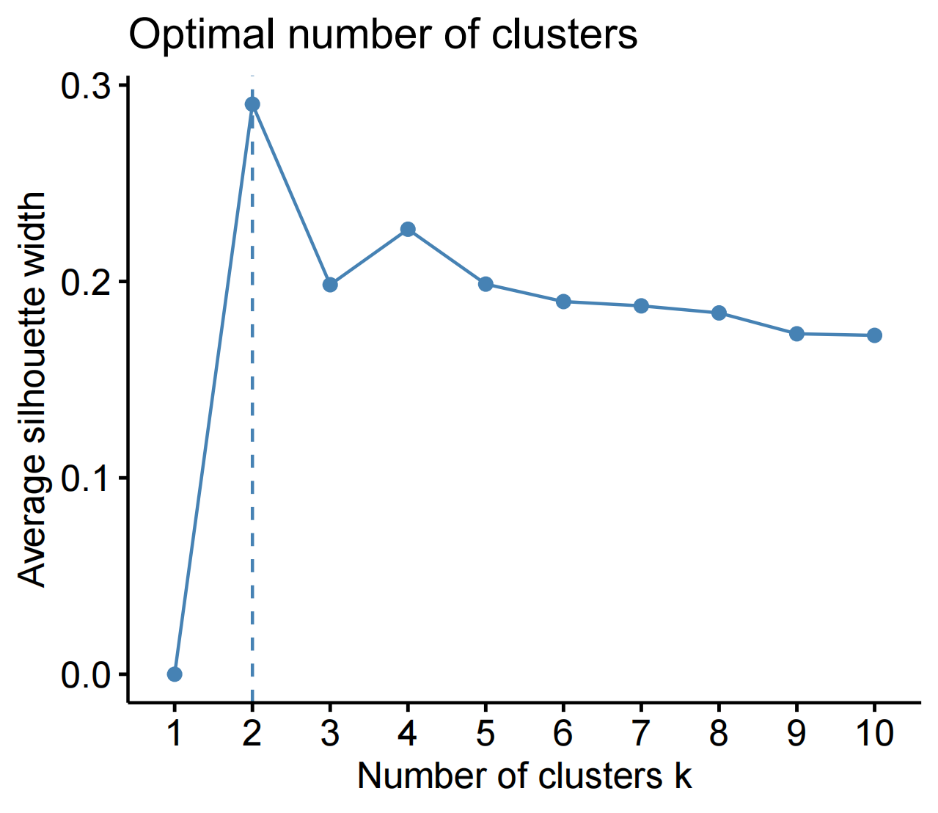


**Supplementary Fig. 1** Silhouette coefficient relationship graph

**Supplementary Table 1** Statistics on missing variables

| **Variables** | **Deficiencies** | |
| --- | --- | --- |
|  | **N** | **%** |
| Age | 0 | 0.00% |
| Education level | 0 | 0.00% |
| Sex | 0 | 0.00% |
| Residence | 0 | 0.00% |
| Marital status | 0 | 0.00% |
| Uric acid | 0 | 0.00% |
| Hypoglycemic drugs | 0 | 0.00% |
| Antihypertensive drugs | 0 | 0.00% |
| Lipid-lowering drugs | 0 | 0.00% |
| Smoking status | 1 | 0.02% |
| Fasting plasma glucose | 4 | 0.07% |
| Hypertension | 21 | 0.39% |
| Glycated hemoglobin A1c | 33 | 0.61% |
| Diabetes | 40 | 0.74% |
| Dyslipidemia | 100 | 1.86% |
| Weight | 596 | 11.09% |
| Height | 612 | 11.39% |
| Drinking status | 1380 | 25.69% |
| Systolic blood pressure | 1541 | 28.69% |
| Diastolic blood pressure | 1543 | 28.72% |

**Supplementary Table 2** Subgroup analysis of the association between Cum-RC score and CVD risk based on tertiles

| **Subgroup** |  | **Cum-RC, OR (95%CI)** | | | **P for trend** | **P for interaction** |
| --- | --- | --- | --- | --- | --- | --- |
|  | **N** | **T1(≤57.36)** | **T2(57.36-87.52)** | **T3(>87.52)** |  |  |
| Age |  |  |  |  |  | 0.928 |
| < 60 | 3145 | 1 | 1.21 (0.90, 1.64) | 1.24 (0.89, 1.74) | 0.169 |  |
| ≥ 60 | 2227 | 1 | 1.15 (0.87, 1.53) | 1.29 (0.93, 1.79) | 0.122 |  |
| Sex |  |  |  |  |  | 0.068 |
| Male | 2468 | 1 | 1.25 (0.92, 1.70) | 1.73 (1.29, 2.34) | 0.002 |  |
| Female | 2904 | 1 | 1.25 (0.95, 1.66) | 1.22 (0.93, 1.61) | 0.974 |  |
| Residence |  |  |  |  |  | 0.432 |
| Rural | 2569 | 1 | 1.32 (0.97, 1.79) | 1.42 (1.03, 1.96) | 0.035 |  |
| Urban | 2803 | 1 | 1.07 (0.80, 1.44) | 1.18 (0.87, 1.60) | 0.278 |  |
| Marital status |  |  |  |  |  | 0.956 |
| Married | 4855 | 1 | 1.17 (0.94, 1.46) | 1.25 (0.99, 1.58) | 0.060 |  |
| Other | 517 | 1 | 1.17 (0.59, 2.29) | 1.47 (0.72, 3.02) | 0.254 |  |
| Smoke |  |  |  |  |  | 0.092 |
| Yes | 2050 | 1 | 1.30 (0.93, 1.83) | 1.76 (1.24, 2.51) | 0.002 |  |
| Never | 3322 | 1 | 1.07 (0.82, 1.40) | 1.03 (0.78, 1.36) | 0.879 |  |
| Drink |  |  |  |  |  | 0.065 |
| Yes | 1225 | 1 | 1.06 (0.68, 1.65) | 1.95 (1.27, 3.01) | 0.002 |  |
| Never | 4147 | 1 | 1.21 (0.95, 1.54) | 1.13 (0.87, 1.45) | 0.388 |  |
| BMI |  |  |  |  |  | 0.090 |
| < 24 | 3172 | 1 | 1.29 (0.99, 1.69) | 1.54 (1.15, 2.06) | 0.004 |  |
| ≥ 24 | 2200 | 1 | 0.95 (0.67, 1.33) | 0.98 (0.71, 1.36) | 0.979 |  |
| Hypertension |  |  |  |  |  | 0.762 |
| Yes | 4238 | 1 | 1.13 (0.89, 1.44) | 1.28 (0.99, 1.66) | 0.062 |  |
| Never | 1134 | 1 | 1.35 (0.89, 2.07) | 1.28 (0.83, 1.95) | 0.347 |  |
| Dyslipidemia |  |  |  |  |  | 0.218 |
| Yes | 413 | 1 | 0.88 (0.42, 1.84) | 0.82 (0.40, 1.68) | 0.615 |  |
| Never | 4959 | 1 | 1.20 (0.96, 1.49) | 1.32 (1.05, 1.67) | 0.019 |  |
| Diabetes |  |  |  |  |  | 0.051 |
| Yes | 255 | 1 | 0.45 (0.14, 1.44) | 1.45 (0.49, 4.29) | 0.159 |  |
| Never | 5117 | 1 | 1.21 (0.98, 1.50) | 1.22 (0.98, 1.53) | 0.083 |  |

In multivariate models, potential confounders other than grouping variables were adjusted for, including age, sex, education level, marital status, residence, BMI, smoking status, drinking status, SBP, DBP, hypertension, dyslipidemia, diabetes, lipid-lowering drugs, antihypertensive drugs, hypoglycemic drugs, FPG, HbA1c, and UA.

**Supplementary Table 3** Multiple logistic regression analysis of Cum-RC and CVD risk after exclusion of extreme BMI

|  | **No. Events (%)** | **Crude** | **P value** | **Model 1** | **P value** | **Model 2** | **P value** | **Model 3** | **P value** |
| --- | --- | --- | --- | --- | --- | --- | --- | --- | --- |
|  |  | **OR (95% CI)** |  | **OR (95% CI)** |  | **OR (95% CI)** |  | **OR (95% CI)** |  |
| **Cardiovascular disease** | |  |  |  |  |  |  |  |  |
| **Cum-RC** |  |  |  |  |  |  |  |  |  |
| Class 1 | 275 (13.62) | 1(Ref) |  | 1(Ref) |  | 1(Ref) |  | 1(Ref) |  |
| Class 2 | 307 (10.44) | 0.74 (0.62,0.88) | 0.001 | 0.76 (0.64,0.91) | 0.003 | 0.78 (0.66,0.94) | 0.007 | 0.78 (0.65,0.94) | 0.008 |
| **Cum-RC** |  |  |  |  |  |  |  |  |  |
| ≤57.36 | 166 (9.73) | 1(Ref) |  | 1(Ref) |  | 1(Ref) |  | 1(Ref) |  |
| 57.36-87.52 | 202 (12.12) | 1.28 (1.03,1.59) | 0.026 | 1.26 (1.01,1.57) | 0.038 | 1.20 (0.96,1.49) | 0.109 | 1.20 (0.96,1.49) | 0.112 |
| >87.52 | 214 (13.49) | 1.45 (1.17,1.79) | 0.001 | 1.46 (1.17,1.81) | 0.001 | 1.31 (1.04,1.64) | 0.019 | 1.32 (1.05,1.67) | 0.017 |
| P for trend |  | 0.001 |  | 0.001 |  | 0.02 |  | 0.018 |  |
| **Heart Disease** |  |  |  |  |  |  |  |  |  |
| **Cum-RC** |  |  |  |  |  |  |  |  |  |
| Class 1 | 177 (8.77) | 1(Ref) |  | 1(Ref) |  | 1(Ref) |  | 1(Ref) |  |
| Class 2 | 185 (6.29) | 0.70 (0.56,0.87) | 0.001 | 0.74 (0.60,0.92) | 0.008 | 0.76 (0.61,0.94) | 0.013 | 0.76 (0.61,0.95) | 0.014 |
| **Cum-RC** |  |  |  |  |  |  |  |  |  |
| ≤57.36 | 105 (6.15) | 1(Ref) |  | 1(Ref) |  | 1(Ref) |  | 1(Ref) |  |
| 57.36-87.52 | 126 (7.56) | 1.25 (0.95,1.63) | 0.107 | 1.21 (0.92,1.58) | 0.169 | 1.16 (0.89,1.53) | 0.273 | 1.17 (0.89,1.54) | 0.256 |
| >87.52 | 131 (8.26) | 1.37 (1.05,1.79) | 0.020 | 1.31 (1.00,1.72) | 0.047 | 1.21 (0.91,1.59) | 0.188 | 1.26 (0.94,1.67) | 0.117 |
| P for trend |  | 0.020 |  | 0.047 |  | 0.192 |  | 0.118 |  |
| **Stroke** |  |  |  |  |  |  |  |  |  |
| **Cum-RC** |  |  |  |  |  |  |  |  |  |
| Class 1 | 116 (5.75) | 1(Ref) |  | 1(Ref) |  | 1(Ref) |  | 1(Ref) |  |
| Class 2 | 135 (4.59) | 0.79 (0.61,1.02) | 0.069 | 0.78 (0.60,1.02) | 0.065 | 0.81 (0.62,1.05) | 0.115 | 0.81 (0.62,1.05) | 0.113 |
| **Cum-RC** |  |  |  |  |  |  |  |  |  |
| ≤57.36 | 66 (3.87) | 1(Ref) |  | 1(Ref) |  | 1(Ref) |  | 1(Ref) |  |
| 57.36-87.52 | 86 (5.16) | 1.35 (0.97,1.88) | 0.072 | 1.36 (0.98,1.89) | 0.069 | 1.27 (0.91,1.77) | 0.163 | 1.26 (0.90,1.76) | 0.180 |
| >87.52 | 99 (6.24) | 1.65 (1.20,2.28) | 0.002 | 1.79 (1.30,2.48) | <0.001 | 1.57 (1.12,2.20) | 0.009 | 1.55 (1.10,2.18) | 0.013 |
| P for trend |  | 0.002 |  | <0.001 |  | 0.008 |  | 0.013 |  |

Cum-RC, cumulative RC; CVD, cardiovascular disease; BMI body mass index; OR, odds ratio; CI, confidence interval; Ref, reference. Crude: unadjusted; Model 1: corrected for age, sex, education level, marital status and residence; Model 2: Model 1+BMI, smoking status, drinking status, SBP and DBP; Model 3: Model 2+hypertension, dyslipidemia, diabetes, lipid-lowering drugs, antihypertensive drugs, hypoglycemic drugs, FPG, HbA1c, and UA.

**Supplementary Table 4** Multiple logistic regression analysis of Cum-RC and CVD risk after exclusion of dyslipidemic population

|  | **No. Events (%)** | **Crude** | **P value** | **Model 1** | **P value** | **Model 2** | **P value** | **Model 3** | **P value** |
| --- | --- | --- | --- | --- | --- | --- | --- | --- | --- |
|  |  | **OR (95% CI)** |  | **OR (95% CI)** |  | **OR (95% CI)** |  | **OR (95% CI)** |  |
| **Cardiovascular disease** | |  |  |  |  |  |  |  |  |
| **Cum-RC** |  |  |  |  |  |  |  |  |  |
| Class 1 | 277 (13.93) | 1(Ref) |  | 1(Ref) |  | 1(Ref) |  | 1(Ref) |  |
| Class 2 | 313 (11.08) | 0.77 (0.65,0.91) | 0.003 | 0.79 (0.66,0.94) | 0.008 | 0.81 (0.68,0.96) | 0.017 | 0.81 (0.67,0.96) | 0.018 |
| **Cum-RC** |  |  |  |  |  |  |  |  |  |
| ≤57.36 | 158 (9.92) | 1(Ref) |  | 1(Ref) |  | 1(Ref) |  | 1(Ref) |  |
| 57.36-87.52 | 200 (12.51) | 1.30 (1.04,1.62) | 0.021 | 1.28 (1.02,1.59) | 0.031 | 1.23 (0.98,1.54) | 0.068 | 1.21 (0.97,1.52) | 0.092 |
| >87.52 | 232 (14.30) | 1.51 (1.22,1.88) | <0.001 | 1.52 (1.22,1.89) | <0.001 | 1.39 (1.11,1.74) | 0.004 | 1.38 (1.09,1.74) | 0.006 |
| P for trend |  | <0.001 |  | <0.001 |  | 0.004 |  | 0.007 |  |
| **Heart Disease** |  |  |  |  |  |  |  |  |  |
| **Cum-RC** |  |  |  |  |  |  |  |  |  |
| Class 1 | 177 (8.90) | 1(Ref) |  | 1(Ref) |  | 1(Ref) |  | 1(Ref) |  |
| Class 2 | 182 (6.44) | 0.70 (0.57,0.87) | 0.001 | 0.74 (0.59,0.92) | 0.007 | 0.75 (0.60,0.94) | 0.011 | 0.74 (0.59,0.92) | 0.008 |
| **Cum-RC** |  |  |  |  |  |  |  |  |  |
| ≤57.36 | 99 (6.22) | 1(Ref) |  | 1(Ref) |  | 1(Ref) |  | 1(Ref) |  |
| 57.36-87.52 | 121 (7.57) | 1.23 (0.94,1.63) | 0.133 | 1.20 (0.91,1.58) | 0.204 | 1.16 (0.88,1.53) | 0.297 | 1.16 (0.88,1.54) | 0.287 |
| >87.52 | 139 (8.57) | 1.41 (1.08,1.85) | 0.011 | 1.37 (1.04,1.79) | 0.023 | 1.27 (0.96,1.67) | 0.096 | 1.32 (0.99,1.76) | 0.057 |
| P for trend |  | 0.011 |  | 0.023 |  | 0.097 |  | 0.057 |  |
| **Stroke** |  |  |  |  |  |  |  |  |  |
| **Cum-RC** |  |  |  |  |  |  |  |  |  |
| Class 1 | 119 (5.99) | 1(Ref) |  | 1(Ref) |  | 1(Ref) |  | 1(Ref) |  |
| Class 2 | 145 (5.13) | 0.85 (0.66,1.09) | 0.201 | 0.84 (0.65,1.08) | 0.174 | 0.87 (0.67,1.12) | 0.269 | 0.87 (0.67,1.13) | 0.287 |
| **Cum-RC** |  |  |  |  |  |  |  |  |  |
| ≤57.36 | 65 (4.08) | 1(Ref) |  | 1(Ref) |  | 1(Ref) |  | 1(Ref) |  |
| 57.36-87.52 | 89 (5.57) | 1.38 (1.00,1.92) | 0.051 | 1.40 (1.00,1.94) | 0.047 | 1.34 (0.97,1.87) | 0.080 | 1.30 (0.93,1.82) | 0.119 |
| >87.52 | 110 (6.78) | 1.71 (1.25,2.34) | 0.001 | 1.82 (1.33,2.51) | <0.001 | 1.67 (1.20,2.31) | 0.002 | 1.58 (1.12,2.21) | 0.008 |
| P for trend |  | 0.001 |  | <0.001 |  | 0.002 |  | 0.008 |  |

Cum-RC, Cumulative RC; CVD, cardiovascular disease; OR, odds ratio; CI, confidence interval; Ref, reference. Crude: unadjusted; Model 1: corrected for age, sex, education level, marital status and residence; Model 2: Model 1+BMI, smoking status, drinking status, SBP and DBP; Model 3: Model 2+hypertension, diabetes, lipid-lowering drugs, antihypertensive drugs, hypoglycemic drugs, FPG, HbA1c, and UA.
